# The social impact of emotional tears

**DOI:** 10.1007/s11031-016-9543-0

**Published:** 2016-02-08

**Authors:** Ad J. J. M. Vingerhoets, Niels van de Ven, Yvonne van der Velden

**Affiliations:** Department of Medical and Clinical Psychology, Tilburg University, P.O. Box 90153, 5000 LE Tilburg, The Netherlands; Department of Social Psychology, Tilburg University, P.O. Box 90153, 5000 LE Tilburg, The Netherlands

**Keywords:** Tears, Crying, Empathy, Prosocial behavior, Personality, Friendliness, Social connectedness

## Abstract

The question what specific functions the production of emotional tears fulfills has received only limited attention of behavioral scientists. We report the results of two studies on the social impact of emotional tears. In Study 1 (96 Dutch females), perceived helplessness and felt connectedness predicted the willingness to help a person depicted as crying tearfully, while perceived friendliness did not. In Study 2 (US sample, 128 males, 68 females) all three of these variables mediated the effect the display of tears had on the willingness to help. Our results replicate and extend previous work and add to current knowledge by showing that tearful crying facilitates helping behavior and by identifying reasons *why* people are more willing to help criers. These findings help to put forth novel predictions on the impact of tearful crying on others.

## Introduction

Tears have fascinated humankind for ages. Classic scholars discussed several intriguing questions, including where tears originate from (the heart or the brain?), how individual and gender differences could be explained, and why crying brings relief (Petitus 1661; Horstmanshoff 2014). Charles Darwin (1872) discussed tearful crying in a more modern, scientific way in his seminal work *The Expression of Emotions in Man and Animals*. Darwin not only connected emotional tears to suffering and distress but also to tender feelings. In addition, he addressed some important developmental aspects of tearful crying and devoted attention to cross-cultural issues. He further acknowledged that basal (i.e., non-emotional) tears serve important functions like lubrication, nourishment, and protection of the eye, whereas the vocal crying of infants solicits the attention of caregivers. However, he saw weeping (i.e., emotional tears) as an incidental result, “as purposeless as the secretion of tears from a blow outside the eye, or as a sneeze from the retina being affected by a bright light…” (Darwin 1872, p. 175). Recently, Darwin’s view that emotional tears do not serve any function is challenged and several hypotheses claiming a role for emotional tears in human evolution have been formulated (e.g., Hasson 2009; Murube 2009; Provine 2012; Trimble 2012; Vingerhoets 2013; Walter 2006).

In the current literature on adult crying (see Vingerhoets 2013; Vingerhoets and Bylsma 2015), two possible major functions of crying have been postulated: (1) catharsis and emotional recovery (an “intra-individual” function) and (2) signaling to others one’s need for support and succor, which results in a change in their ongoing behavior and in the directing of their attention to the crier (the “inter-individual” function). The main theories that address the interpersonal functions of crying (e.g., Bowlby 1969; Hasson 2009; Nelson 2005; Vingerhoets 2013; Walter 2006) hypothesize that crying elicits helping behavior in others. However, this hypothesis has not yet been firmly empirically established in adults. We, therefore, test the basic hypothesis that crying elicits helping behavior in others. Furthermore, we explore the possible underlying mechanisms.

## Why crying might elicit helping behavior

Three reasons have been given why crying might elicit helping behavior. The first is the argument provided by, amongst others, Provine et al. (2009), who demonstrated that someone who is tearful is seen as sadder than someone without tears. As sadness and helplessness are closely related, we expected that the display of tears would increase the perceived helplessness of a person, which in turn leads to a higher willingness to help that person (see also Horstmann 2003). Throughout ages, from the seventeenth century philosopher Thomas Hobbes to, more recently, emotion psychologist Frijda (1986) and evolutionary biologist Hasson (2009), scholars have emphasized that it is, in particular, helplessness as well as loss and separation that are the main antecedents of tears all over the life span (Nelson 2005; Vingerhoets 2013; Vingerhoets et al. 1997 for empirical findings).

A second reason why crying triggers helping behavior is that crying individuals are typically perceived as more agreeable and less aggressive (Hasson 2009; Hendriks et al. 2008) and that crying individuals elicit more sympathy and compassion (Zeifman and Brown 2011). These findings suggest that tearful individuals might also be more positively evaluated and be perceived as more friendly. Aggression and agreeableness (the Big-Five personality trait that reflects someone’s friendly and likable nature; Costa and McCrae 1992) are negatively related (Gleason et al. 2004). Furthermore, being sympathetic is a key aspect of being an agreeable person (Gosling et al. 2003). People thus might be more willing to help a person who is crying because they perceive him or her as more agreeable and friendly.

A third (and related) explanation why tears might elicit helping behavior is that seeing tears might make us feel more closely connected to the crying individual. This increase in felt connectedness with a crying individual could also promote prosocial behavior. In other words, the closer we feel to another individual, the more altruistically we behave towards that person (e.g., Bressan et al. 2009). It is tempting to refer here also to ritual weeping as a means to promote social bonding (e.g., Dissanayake 2008). In the anthropological literature, several examples can be found of ritual or common weeping after adversity and disasters or when preparing for war, all suggesting that common tears forge bonds between people (see Vingerhoets 2013).

Empirical support for the notion that tears have a strong signal value has been provided in several previous studies. Visible tears may make others believe that the person is sad and helpless (Balsters et al. 2013; Cornelius and Lubliner 2003; Cornelius et al. 2000; Hendriks and Vingerhoets 2006; Provine et al. 2009; Zeifman and Brown 2011). The digital removal of tears of a picture of a crying individual revealed that tears not only facilitate the perception of sadness, they also seem to resolve the ambiguity of facial expression. More precisely, the digital removal of tears of crying individuals produced faces of uncertain emotional valence—varying from awe, concern, contemplation, fright, puzzlement to, occasionally, sadness (Provine et al. 2009).

## The present studies

Given that the presence of visible tears seems to help to clarify the possible ambiguity of a facial expression and that visible tears emphasize the need for help and succor, the here reported studies were designed specifically to obtain more insight into the link between visible tears and the willingness to provide support.

The two here reported studies are the first to test whether visual tears elicit more helping behavior in others, and especially also why this is the case. Is it because people consider crying individuals as more in need of help, as more friendly, or because one feels more socially connected to them, or a combination of factors?

Examining why people are willing to help those who cry will increase our understanding of the functions of crying. For example, if people help those who cry more because they are perceived as sad and helpless, it can be expected that those who are seen as powerful might benefit less from displaying tears than those who we consider as weak. If, on the other hand, people are more willing to help those who cry because they feel more connected to them, this would lead to the prediction that crying has stronger effects for those we can more easily feel connected to, such as people who are relatively similar to ourselves, or with whom we have a special relationship.

In conclusion, the two current studies were designed to replicate and extend previous research findings concerning the effects of visible tears on person evaluation and the willingness to provide support (Balsters et al. 2013; Cornelius and Lubliner 2003; Cornelius et al. 2000; Provine et al. 2009; Zeifman and Brown 2011). We evaluated whether visible tears make people not only be perceived as more sad and helpless, but also as more friendly, and make observers feel more connected to them. Finally, and crucially, we examined which specific attributed features mediate the effect of tears on the reported willingness to help the tearful individual.

## Study 1

### Methods

#### Participants

Ninety-seven Dutch female first-year psychology students, who received course credits for participation, took part in the study. We removed data of one participant, because of her older age (48 years), compared to the age distribution of the other participants. Consequently, data of 96 women, aged 17–36 (M = 19.30, SD = 2.27) were included in the analyses.

#### Pictures

The visual stimuli used in the experiment consisted of 40 full color 18 by 18-in. photos. Half of them were pictures of a person crying with visible tears; the other half consisted of the same pictures with the tears digitally removed. The photos were preselected in a pilot study from a set of 26 pictures.^1^ The original 20 photos depicted 14 women and 6 men. From the 40 final photos (20 with tears and 20 with the tears removed) we created two different sets of 20 pictures (10 with and 10 without tears), with an equal gender balance of the depicted persons. Participants were exposed to one of these sets, to prevent them from rating pictures of the same individual with and without tears.

This study was part of a larger exploratory study that also tested the effect of background music on the evaluation of faces. Participants wore headphones, although the here presented data were from the no music condition. The experiment was run on a PC with a 17-in. CRT monitor. A specially designed response box was used to record the responses. Participants were tested in pairs with their backs turned to each other sitting on a normal office chair at a table approximately 25 in. from the monitor.

#### Dependent variables

Nine statements with a rating scale from 1 (= I do not agree with the statement) to 9 (= full confirmation of the statement) were used to assess the participants’ evaluations of the person and how they would respond to the displayed person when they would encounter him or her. The nine statements represented four dimensions: helplessness, friendliness, connectedness, and willingness to help.^2^ Perceived *helplessness* (α = .91) of the depicted individual was measured with two items, “This person needs support” and “This person needs consolation.” Perceived *friendliness* (α = .88) of the depicted individual was measured with the following two items “This person seems kind” and “This person seems likable.” *Connectedness* (α = .82) with the depicted individual was measured with three items “I feel connected to this person”, “I feel emotionally involved with this person” and “I sympathize with this person”, and the single item of the Inclusion of Other in the Self Scale (IOS; Aron et al. 1992), which comprises a set of Venn-like diagrams with different degrees of overlap of two circles that refer to how close one feels to the depicted person. The final construct, *willingness to help* (α = .93) the depicted individual, was evaluated with two items; “I am inclined to ask this person whether I can be of any help” and “I am inclined to comfort this person.” The statements were presented in a fixed, random order.

#### Design

The study applied a within-subjects design. Each participant evaluated both pictures of individuals with visible tears and pictures of individuals with the tears digitally removed.

#### Procedure

Participants started with reading the instruction, followed by informed consent and training trials, in which they saw a picture of a person (without tears) for 5 s and were asked to rate the depicted person on three statements (for which they had a maximum of 15 s to respond). In the main task, each stimulus presentation was the picture of a person with or without visible tears. The participants were asked to respond as quickly as possible to the evaluation questions. Each picture was displayed three times, followed by three (out of the nine) subsequent rating statements. Completion of the task took approximately 25 min.

### Results

For each participant, we calculated mean values of the ratings of the pictures of people with and without tears.^3^ Table 1 displays the descriptive statistics and the results of the statistical comparisons, Table 2 contains the correlation matrix of the variables (separated for pictures with and without tears).

**Table 1:** Participants’ responses towards pictures with tears and pictures on which the tears had been digitally removed (Study 1)

	No tear		Tear		Statistics		
	M	(SD)	M	(SD)	Paired-*t*	*p*	*d*
Helplessness	5.21	(1.21)	6.93	(1.08)	13.43	<.001	1.36
Friendliness	5.66	(1.09)	6.11	(0.91)	5.35	<.001	0.57
Social connectedness	3.75	(1.11)	4.55	(1.30)	9.77	<.001	1.00
Willingness to help	4.38	(1.30)	5.90	(1.51)	12.96	<.001	1.32

Answer scale from 1 to 9

**Table 2:** Correlations between the variables, for Tears/No Tears, respectively (Study 1)

	Friendliness	Social connectedness	Willingness to help
Helplessness	.57/.46	.46/.63	.65/.78
Friendliness		.45/.51	.51/.49
Social connectedness			.74/.80

All correlations significant at *p* < .001

As Table 1 shows, for all four dependent variables, we find strong effects of the presence of visible tears. Tearful individuals were regarded as more helpless, more friendly, and participants reportedly felt more socially connected with them. Importantly, participants also indicated to be more willing to provide help to a tearful than to a non-tearful person.

These pairwise comparisons are also the first step of a mediational process analysis for a within-subjects design (Judd et al. 2001). First, to establish if there is a mediation in a within-subjects design, the pairwise t-tests comparing the condition effect on the dependent variable (willingness to help) and the mediators (perceived helplessness, friendliness, and connectedness) should both be significant, as is the case in the present data. Second, the between conditions difference score of the possible mediator under investigation should predict the difference score of the dependent variable. For example, in our case, the display of tears is expected to increase both the perceived helplessness of the crying person and the willingness to help. In addition, the increase in perceived helplessness should predict the increase in the willingness to help.

To test this latter step of the mediation analysis, a multiple regression analysis was conducted. We added the difference scores between participants’ ratings of the pictures with and without tears on perceived helplessness, friendliness, and social connectedness as predictors of the difference score in the willingness to help. The regression model was strongly significant, *F*(3,92) = 54.24, *p* < .001, adj-R^2^ = .63. The increases in perceived helplessness, *b* = 0.46, *se* = 0.07, β = .51, *t* = 7.17, *p* < .001, and social connectedness, *b* = 0.64, *se* = 0.11, β = .45, *t* = 5.66, *p* < .001, due to the presence of tears predicted the increase in willingness to help, whereas the increase in perceived friendliness did not, *b* = −0.06, *se* = 0.10, β = −.04, *t* = 0.58, *p* = .56. This pattern of findings confirms that the display of tears increases the willingness to help and that this effect is mediated by an increased perceived helplessness due to the visibility of tears and to a more strongly felt connectedness to an individual in tears. Although there was also an increase in perceived friendliness of someone who was crying, this did not lead to an increased willingness to help.

### Discussion

In Study 1 we replicated and extended earlier findings showing that visible tears make people not only look more sad and helpless but also more friendly and observers feel more connected with those persons. Importantly, corroborating previous research (Balsters et al. 2013; Provine et al. 2009), we also found that people reported a greater willingness to help tearful persons. Additionally, we demonstrated that especially the perceived helplessness of the tearful person and the increased social connectedness induced by the tears seems to make people more likely to help the crying person. The higher perceived friendliness of a tearful person, in contrast, failed to predict helping intentions in this study.

## Study 2

We designed Study 2 to cross-validate and extend the previous findings. First, we used a between-subjects design instead of a within-subjects design, which also allowed us to perform a mediation analysis. Second, since previous research suggested that men and women might differ in their reactions to crying individuals (see Vingerhoets and Bylsma 2015, for a review), with men having a greater tendency to react with confusion and helplessness, we now include both men and women as participants (who were also from a different country than in Study 1).

### Methods

#### Participants and design

We aimed to include 200 participants via Amazon Mturk (see Paolacci and Chandler 2014) and eventually obtained 196 (128 males, 68 females. *M*_age_ = 31.85 years, SD = 10.24, range 18–67). Participation was limited to US based Mturk volunteers with >95 % acceptance rate of their work, who received $0.40 for their participation. The volunteers were randomly assigned to a condition in which they saw a picture (from the same picture set as used in Study 1) of a woman with visible tears (Tear condition, N = 98) or the same woman with the tears digitally removed (No Tear condition, N = 98).

#### Procedure

Participants were instructed to imagine that they encountered the—tearful or not tearful—depicted woman. Subsequently, they answered three questions for *social connectedness* (α = .90): “I would feel connected to her”, “I would feel emotionally involved with her” [on scales from 1 (not at all) to 7 (very much so)], and the IOS (Aron et al. 1992) that uses circles to depict interpersonal closeness (from 0 to 6, with higher scores indicating closer feelings; Aron et al. 1992). Participants additionally indicated how *helpless* they thought the person looked, using two items (α = .79): “How sad do you think she is?” and “How helpless do you think she is?”. Perceived *friendliness* was measured with two items (α = .86): “The person looks like a sympathetic person” and “The person seems like a nice person.” Finally, *willingness to help* was evaluated with two items (α = .91); “If I met her, I would be inclined to ask her if I could help” and “If I met her, I would like to be able to support her.” All these questions were answered on a scale from 0 (not at all) to 6 (very much so). To make the first two questions on connectedness better comparable to the others, we transformed the 1–7 scale used for these questions to the 0–6 scale used for the other questions by subtracting 1 from the original answer. For each construct, we calculated the mean scores of the relevant items as the unit for analysis.

### Results

Table 3 presents the mean ratings per condition for each of the measured constructs and the results of the between groups *t*-tests comparing them. Table 4 contains the correlations between all variables. There were no effects of gender on the dependent variables, nor were there any interaction effects of gender and condition, all *F*’s(1,192) ≤ 2.28, *p*’s ≥ .133, η_p_^2^ ≤ .01.

**Table 3:** Participants’ responses towards pictures with tears and pictures on which the tears had been digitally removed (Study 2)

	No tear		Tear		Statistics		
	M	(SD)	M	(SD)	*t*(194)	*p*	*d*
Helplessness	2.94	(1.49)	4.07	(1.01)	6.17	<.001	0.88
Social connectedness	1.59	(1.32)	2.24	(1.36)	3.45	.001	0.49
Friendliness	3.46	(1.24)	3.96	(0.98)	3.13	.002	0.45
Willingness to help	2.75	(1.76)	4.10	(1.40)	5.95	<.001	0.85

Answer scale from 0 to 6

**Table 4:** Correlations between variables in Study 2

	Friendliness	Social connectedness	Willingness to help
Helplessness	.29	.22	.52
Friendliness		.44	.55
Social connectedness			.49

All correlations significant at *p* ≤ .002

As Table 3 shows, participants clearly perceived the tearful person to be more helpless than when the tears had been removed and also felt more connected to the person. They also rated her as more friendly when she shed tears. Finally and most importantly, participants indicated that they would be more willing to offer the displayed woman help if she is depicted with.

**Fig. 1:**
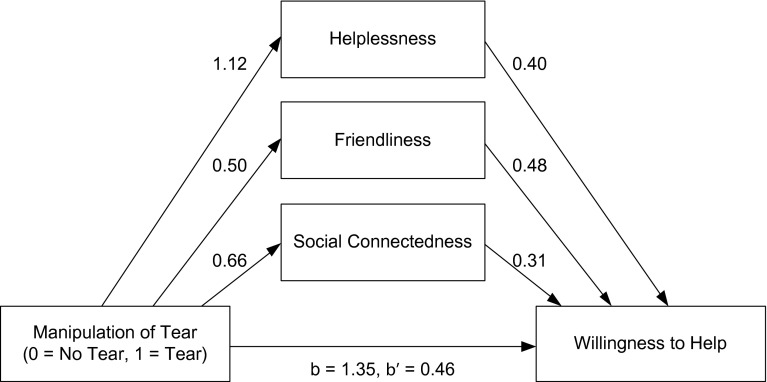
Mediation model summarizing the direct and indirect effects of tears on the willingness to help (Study 2)

We additionally conducted a mediation analysis via bootstrapping following the method of Preacher and Hayes (2008) using 10,000 samples and bias corrected intervals. As Fig. 1 shows, the effect of visible tears on the willingness to help was mediated by all three other constructs. The direct effect of tears on the willingness to help, *b* = 1.35, *se* = 0.23, *t* = 5.95, *p* < .0001, became much weaker when taking into account the indirect effects of the other three variables, *b* = 0.46, *se* = 0.19, *t* = 2.39, *p* = .018. The indirect effects of perceived helplessness (95 %CI 0.20–0.75), felt connectedness (95 %CI 0.08–0.41), and perceived friendliness (95 %CI 0.09–0.46) were all significant \as indicated by the confidence intervals that all excluded 0. This finding confirms that each of these three variables has an effect on the willingness to help, even when we control for the effects of the other variables. When controlling for these other variables, a smaller direct effect of the tears on the willingness to help remained, which suggests that tears have additional effects that promote prosocial behavior.

### Discussion

In this study, we replicated and extended the results found in Study 1 in a different culture and among both genders, using a between-subject rather than a within-subject design. The data confirmed that, on the one hand, the presence of visible tears impacted in particular on the helplessness rating and substantially less on the social connectedness and friendliness ratings. However, on the other hand, perceived friendliness had as strong a relation with the willingness to help as perceived helplessness had. Finally, although all three factors that we examined in the present studies seemed to promote the willingness to help someone who is crying, it is clear that there are still other factors that additionally explain the greater willingness to help.

## General discussion

The here reported studies replicated and extended prior work demonstrating that people evaluate a tearful person as more in need of help and as more friendly. Importantly, people further seem to feel more connected to tearful persons. Of main interest to us was the question whether these person perceptions, in their turn, predicted the willingness to help that person. In other words, do people tend to offer help to a crying person because (s)he is seen as more helpless, as more friendly, or because one feels more connected to him or her? Our results suggest that in particular the perceived helplessness and the increased feeling of being connected facilitate the increased willingness to help tearful individuals. Friendliness was the variable for which the visibility of tears had the smallest effect in both studies, and in Study 1 it even failed to show an association with the willingness to help. These findings are important and novel observations because earlier research had failed to clarify possible reasons why people offer help to tearful individuals.

The present studies corroborate earlier findings that tears not only have a strong impact on the perception of the crying individual but that they also change behavioral intentions of observers. Since our stimuli were identical images of a tearful individual or with the tears being removed digitally, it can be concluded that the mere presence of visible tears facilitates prosocial behavioral intentions. Taken together, the current findings seem compatible with the notion that tears may have contributed to the development of Homo Sapiens as an ultra-social species (e.g., Hasson 2009; Vingerhoets 2013; Walter 2006) by promoting empathy and feelings of being connected, which, in their turn, might facilitate prosocial behavior and mutual collaboration.

Our findings not only confirm that crying elicits helping behavior, but it also allows for new predictions. For example, given the finding that people are willing to help those who cry because they are perceived as helpless, it could be expected that those who are seen as powerful might not or substantially less benefit from displaying tears (if we still perceive them as powerful, they might be regarded as not in need of support).

The finding that tears increase the social connectedness to a person could also imply that tears will have a stronger effect for those we easily feel connected to (e.g., those similar to us, kin and non-kin special relationships), or that they will have a stronger effect in situations in which the link between feeling socially connected and the willingness to help is stronger (e.g., in particular if there is a need of social support). Our present findings can help to come up with new predictions on when crying is more or less effective to elicit helping behavior. The finding that visible tears facilitate feelings of social connectedness seems to support the anthropological hypothesis that common ritual weeping, especially in times of adversities or when preparing for war, is especially meant to promote social bonding (Dissanayake 2008; Vingerhoets 2013).

Note that one should be aware that our findings do not paint the full picture of responses to crying. The present findings cannot obscure that there is ample anecdotal and limited research evidence that crying individuals, just like depressed patients and victims and others expressing suffering do not always elicit sympathy and an increased willingness to provide support (Herbert and Dunkel-Schetter 1992). Crying is also known sometimes to induce irritation, annoyance, and occasionally aggression in observers (see Vingerhoets 2013; Vingerhoets and Bylsma 2015), even in a clinical context (Alexander 2003). For example, infants who cry excessively are at increased risk of physical abuse (Reijneveld et al. 2004), and children who cry easily may be at greater risk of becoming the victim of bullying (Von Salisch 2001). Further, women who cry in rape situations are at increased risk of being physically assaulted (Ullman and Knight 1993; Zoucha-Jensen and Coyne 1993). Also in the work setting, inappropriate tears may have a damaging effect on one’s career (Poverny and Picascia 2009).

Given the great diversity in reactions to crying, further research is needed to identify the specific factors that co-determine the specific nature and intensity of the reactions of observers. To facilitate such research, a model needs to be developed, in which characteristics of the crier and observer, their mutual relationship, the specific antecedents and the felt appropriateness of the crying, and crying characteristics all are taken into account (e.g., Vingerhoets 2013). How important are personality traits or dispositions such as empathy, extraversion, or narcissism? Do insecurely attached individuals perhaps respond differently to crying than their securely attached counterparts? If felt social connectedness is an important mediator of the effect of tear on the willingness to help, perhaps personality traits such as the need to belong also are important for how people respond to someone who cries. And what about the nature of the relationship between the observer and the crier? Does it make a difference when it concerns a patient and his therapist, a supervisor and a worker, two strangers, or two romantic partners? Future research identifying the specific factors that determine the reactions of others to tears has not only important theoretical but also practical relevance.

## Strengths and limitations of the studies

A major strength of the present studies was that we used the same pictures, with and without tears. The mere sight of tears in another individual thus seems to have a strong impact on observers, in particular on perceived helplessness and feelings of social connectedness and prosocial behavioral intentions. An additional strength is that we used samples from different countries and (in the second study) containing both men and women. Moreover, the lack of context is both a strength, because the context-free setting may be considered as a baseline measure how people normally respond to someone who is crying, but at the same time also as a weakness because it is plausible that, among others, the attributed reason for crying—whether it is perceived as appropriate (e.g., the loss of a significant other, physical pain) or most inappropriate (e.g., self-pity, guilt tears)—also strongly determines how people react to tears.

Both studies also have some further important limitations. Since we presented pictures of (non)crying individuals, the focus was solely on the effects of visible tears, without the typical accompanying vocalizations or sobbing, which each may have their separate, additional effects. Until now, crying has been studied mainly as an integral behavior, with no attention to the specific influences of its individual components

We further relied on self-reports, which might be subject to social desirability. In particular, given some discrepancies between the findings of earlier vignette studies (Hendriks et al. 2008) and of a retrospective study on the effects of tears on strangers (Vingerhoets 2013), it is not certain that the self-reported willingness to provide support turns into actual prosocial behavior in real life. Whether or not the prosocial intentions will result in actual behavior is a further research question that deserves the attention of investigators. Since, in evolution and adaptation, only behavior counts, the use of implicit measures and, in particular, behavioral measures is a most desirable extension in future research. Further, it must be realized that the amount of explained variance of the willingness to help was limited, which strongly suggests that other, still to be identified factors, might play an important role as well.

## Conclusion

The here presented two studies have yielded converging evidence that people are more likely to help a person with visible tears than the same person without tears. This effect likely exists (1) because people perceive tearful individuals as more helpless and in need of support, (2) because tears make observers feel more connected with the crying individual and, to a less extent, (3) because tears may individuals also look friendlier. These studies are an important first step toward the solving of the mystery of the social impact of tears, as they experimentally substantiate the notion that adult weeping is also a signal that elicits helping behavior. The studies also form a firm basis to design future studies in which the focus will be on, for example, the effects of context and other possibly relevant factors, and the role of the specific individual components of crying.

## Footnotes

1 See http://www.flickr.com/photos/themuseumofmodernart/sets/72157623741486824.

2 The exact Dutch questions were (in order of presentation): “Deze persoon heeft behoefte aan steun”, “Deze persoon heeft behoefte aan troost”, “Hoe aardig lijkt deze persoon?, “Hoe sympathiek lijkt deze persoon?”, “Ik voel me emotioneel betrokken bij deze persoon”, “In hoeverre leef je mee met deze persoon?”, “In hoeverre voel je verbondenheid met deze persoon?”, “Als ik met deze persoon alleen in een kamer zou zijn dan zou ik vragen hoe het gaat”, and “Als ik met deze persoon alleen in een kamer zou zijn dan zou ik hem/haar troosten”.

3 We checked whether gender of the target person mattered to our participants. We found main effects for all four variables, in that females were seen as more helpless than males and as more friendly, and participants felt more connected to females and wanted to help them more, all *F*’s(1,95) ≥ 5.30, *p* ≤ .024, η_p_^2^ ≥ .05. Only for perceived helplessness was there an interaction of gender with the display of a tear, *F*(1,95) = 4.81, *p* = .031, η_p_^2^ = .05. This interaction means that a female who displayed a tear (*M* = 7.05, SD = 1.19) was perceived slightly more helpless than without the tear (*M* = 5.50, SD = 1.25), compared to this difference for males (*M*_Tear_ = 6.42, SD = 1.72; *M*_NoTear_ = 4.36, SD = 1.61), but the effect was clearly still strong for males, paired-*t*(95) = 10.52, *p* < .001, *d* = 1.07. In other words, the general effect of tears was the same for males and females on three out of four of our measures, and on the fourth one there was a small effect, but a large effect of displaying a tear on the perception of helplessness remained. For further analyses we therefore collapsed across genders. Note that this finding that gender does not appear to matter for the social effect of tears seems at odds with earlier findings of Fischer et al. (2013).
